# Digital intervention targeting nutrition and physical activity behaviours among healthy individuals in low- and middle-income countries: a scoping review

**DOI:** 10.1186/s41043-025-01091-y

**Published:** 2025-10-03

**Authors:** Adi Lukas Kurniawan, Theresia Theresia, Amelia Faradina, Rathi Paramastri, Nila Reswari Haryana, Riska Mayang Saputri Ginting, Bira Arumndari Nurrahma, Larasati Sekar Kinasih, Achmed Forest Khan, Arif Sabta Aji, Zakia Umami, Olivinia Qonita Putri, Noor Rohmah Mayasari

**Affiliations:** 1 Transdisciplinary Research Area “Technology and Innovation for Sustainable Futures” and Center for Development Research (ZEF), Rheinische Friedrich-Wilhelms University of Bonn, Beringstr. 1, 53115 Bonn, Germany; 2 Heidelberg Institute of Global Health (HIGH), Medical Faculty and University Hospital, Heidelberg University, Heidelberg, Germany; 3 Bachelor of Nursing Study Program, Faculty of Medicine, Ganesha University of Education, Buleleng, Indonesia; 4 School of Nutrition and Health Sciences, College of Nutrition, Taipei Medical University, Taipei, Taiwan; 5 Study Program of Nutrition, Faculty of Engineering, State University of Medan, Medan, Indonesia; 6 Department of Nutrition, Faculty of Public Health, Mulawarman University, Samarinda, Indonesia; 7 Department of Biochemistry, Faculty of Medicine, Gadjah Mada University, Yogyakarta, Indonesia; 8 Department of Pharmacology and Nutritional Sciences, College of Medicine, University of Kentucky, Lexington, KY USA; 9 Department of Biomedical Sciences, Faculty of Medicine and Health Sciences, State Islamic University of Maulana Malik Ibrahim, Malang, Indonesia; 10 Department of Nutrition, Sebelas Maret University, Surakarta, Indonesia; 11 Research Center for Biomedical, National Research and Innovation Agency, Bogor, Indonesia; 12 Department of Nutrition, Faculty of Science and Technology, University of Al Azhar Indonesia, Jakarta, Indonesia; 13 Department of Nutrition, Faculty of Sport and Health Sciences, State University of Surabaya, Surabaya, Indonesia

**Keywords:** Digital nutrition intervention, Physical activity, Lifestyle behaviours, mHealth, Scoping review, Low- and middle-income countries

## Abstract

**Background:**

The adoption of digital technologies to enhance health behaviours is on the rise due to their high accessibility. This scoping review summarized evidence on digital interventions designed to improve nutritional status and physical activity (PA) among healthy individuals in low- and middle-income countries (LMICs).

**Methods:**

A comprehensive search of five electronic databases, including MEDLINE via PubMed, Embase, Web of Science, CINAHL, and CENTRAL through the Cochrane Library from January 2000 to September 2024 was conducted. The search strategy was guided by the Participants, Concept, and Context model. Eligible studies included randomized controlled trials and quasi-experimental designs that implemented digital nutrition interventions among healthy individuals in LMICs.

**Results:**

The review included 53 studies, predominantly from Asia, that employed various digital platforms, including social media, text messages, mobile apps, video games, and websites. The interventions primarily targeted children or adolescents and youth, focusing on PA and nutrition-related knowledge, attitudes, and practices. The majority of studies reported significant improvements in PA levels, nutrition knowledge, and healthy food consumption. However, the impact on anthropometric and body composition outcomes was inconsistent might be due to the heterogeneity of intervention, varying duration, diverse target populations, and methodological approaches.

**Conclusion:**

Digital interventions were generally effective in improving PA levels, nutrition knowledge, and healthy food consumption. However, our review identified several gaps, including the limited application of theoretical frameworks and needs assessments in the development of interventions, as well as a narrow focus on specific outcomes. Future studies should expand to diverse regions, incorporate theory-based, context-specific approaches, and adopt double-duty action strategies to enhance the effectiveness and reach of digital nutrition interventions in LMICs.

**Supplementary Information:**

The online version contains supplementary material available at 10.1186/s41043-025-01091-y.

## Introduction

Non-communicable diseases (NCDs) represent a significant and growing public health challenge worldwide, accounting for approximately 82% of global NCD-related deaths, with a disproportionate impact on low- and middle-income countries (LMICs). The rise of NCD-related deaths over the years highlights the urgent need for effective preventive strategies [1, 2]. Focusing on prevention among the healthy population, who might not yet have developed chronic conditions but are at risk due to modifiable lifestyle factors, is recognized as the most effective strategy to lessen the rising burden and reduce the risk of future NCDs [3]. Among the key behavioural risk factors for NCDs, physical inactivity and unhealthy diets are the most prominent factors [4].

Addressing the NCD epidemic requires a multifaceted approach that includes policy interventions, community engagement, and health education to promote healthier behaviours. Community-based programs can also play a crucial role by fostering awareness and encouraging individuals to adopt healthier lifestyles through education and support networks [5]. In recent years, the proliferation of digital technologies such as smartphones, fitness bands, and mHealth platforms has opened new avenues for health promotion [6]. These tools offer innovative methods for educating, informing, and monitoring health behaviours in real-time, potentially reaching a vast audience [7]. Additionally, these technologies can support behavioural interventions by delivering tailored educational content and fostering community support through online platforms. Digital health promotion (DHP) strategies, characterised by their user-centric approach, aim to increase awareness of health habits and support individuals in making health-related decisions [6, 8]. Furthermore, DHP facilitates access to healthcare services, particularly for populations residing in remote or underserved regions [9]. This expansion enhances the availability of health promotion services and provides personalized care. Telemedicine, for example, enables patients to consult healthcare providers without the need for physical visits, thereby reducing barriers to care and improving adherence to treatment plans [10].

The increasing accessibility of digital technologies suggests the potential for improving health behaviours among young and adult populations. Despite the promise of digital interventions, research on their effectiveness in LMICs remains limited [6]. Moreover, significant knowledge gaps persist regarding how digital technology can effectively foster healthy lifestyle behaviours in LMICs and among healthy individuals [6]. This focus is crucial for identifying effective preventive measures and informing public health initiatives aimed at reducing NCDs incidence prior to the onset of chronic conditions, differentiating it from interventions designed for disease management within the clinical population. Therefore, this scoping review aimed to summarize evidence on digital interventions designed to improve nutritional status and physical activity (PA) in LMICs settings. We also explored the effectiveness and gaps of various digital nutrition and physical activity strategies, including social media platforms, mobile and web apps, and AI-powered chatbots, in promoting healthy behaviours.

## Methods

### Data sources, search terms, and search strategy

As a key aspect of our main approach, we conducted a comprehensive search of electronic databases, including MEDLINE via PubMed, Embase, Web of Science, CINAHL, and CENTRAL through the Cochrane Library, spanning a period from 01 January 2000 to 30 September 2024. This period was selected to capture rapid development and increasing adoption of digital technologies in health interventions in LMICS and ensure comprehensive coverage of all relevant studies published since digital tools and platforms became widely accessible. We utilized a combination of MeSH terms and text words related to digital nutrition interventions to identify potentially relevant published studies. Guided by the Participants, Concept, and Context (PCC) model (Table 1), we formulated a targeted search strategy. This strategy employed indexing terms such as MeSH terms, keywords, and free text words. We adapted the PubMed strategy (Supplementary File 1) to suit the other databases. For each search, we documented the following details: databases searched, date of search, search strategy (including any expanded, truncated, or combined terms), filters applied, and the number of records retrieved. Additionally, we provided a source for each publication identified through a manual search (such as journal name, website, or conference proceedings). Our protocol was registered prospectively on Open Science Framework (https://osf.io/ba8cv) based on PRISMA-ScR.

**Table 1:** Participants, concept, and context (PCC) model for the scoping review

Item	Criteria
Participants	Studies involving healthy participants from LMICs without any chronic diseases
Concept	Studies that incorporate one or more of the interventions: nutrition education, physical education, promoting healthy diets and/or PA, and nutritional counselling
Context	Studies involving digital intervention include interventions using mobile applications, digital applications, internet platforms, video games, and social media applications
Type of sources	Randomized controlled trials, quasi-experimental studies, including controlled before-after studies

### Eligibility criteria

The following criteria were used for inclusion: (1) original, peer-reviewed articles written in English, (2) interventions that utilized digital platforms such as online, social media, the internet, video games, and/or mobile applications as the main intervention education platform to improve nutrition status, nutrition behaviour, nutrition knowledge, nutrition literacy, and PA, (3) randomized controlled trials (RCTs) with individual or cluster randomization, or quasi-experimental studies including before-after studies with comparison groups, and (4) studies targeting healthy individuals without chronic diseases (e.g. diabetes, cancer, hypertension, cardiovascular diseases, coronary heart diseases, etc.) aged 10 or older living in LMICs—according to the World Bank’s classification [11]. We selected this threshold based on WHO standards for age-group distributions, which include the full span of adolescence (10–19 years) [12] and adulthood (≥ 20 years)—a period characterized by increasing autonomy in health-related behaviours and greater engagement with digital technology. Studies with a broader reported age range were included if they explicitly contained a sub-population or a significant portion of participants aged ≥ 10 years. Studies targeting overweight or obese individuals, studies targeting individuals “at risk” of developing any chronic diseases, and studies without comparator groups or controlled before-after studies that did not account for baseline differences between study arms were excluded, such as editorials, commentaries, opinions, review articles, descriptive or observational studies, cross-sectional designs, cohort studies, and case–control studies. However, review articles were utilized to identify additional original articles. Furthermore, this review excluded studies that incorporated only wearable device technology as the primary intervention platform without integrating a digital education component.

### Data management

The electronic database records were imported into Covidence (Veritas Health Innovation, Melbourne, Australia), a web-based systematic review management tool. Covidence was used for the detection and removal of duplicates, title and abstract screening, and full-text screening.

### Selection of studies

Two independent reviewers screened the titles, abstracts, and full texts of the studies, excluding those that were irrelevant based on the predetermined eligibility criteria. In the event of any disagreements or conflicts of opinion, the reviewers resolve them through discussion, and if necessary, consult a third party for a final decision.

### Data extraction

A data extraction form was developed in Covidence and two independent reviewers extracted and entered data from the included studies. The following information was extracted from the study: (1) study details including title, authors, journal name, calendar year of publication, and country; (2) study methods including type of study design, sample size, and sample characteristics (i.e., age and sex); (3) intervention strategy including delivery platform, contents of the intervention, duration, frequency, conceptual framework used to develop the intervention, and the comparator group; (4) findings including the effectiveness of the intervention, and theory to explain success and failure.

### Summary of evidence

In this summary of evidence, we documented information on the authors, countries, participants' characteristics, intervention contents, mode of delivery platform, comparators, and a summary of findings. The quantitative studies were summarized by the number of studies that assessed nutritional or PA outcomes, along with the number of studies that demonstrated evidence of an effect on these outcomes. The presentation of qualitative studies was structured around five central themes, including the assessment of acceptability, satisfaction, and perceived effectiveness of the intervention, as well as an exploration of the challenges encountered during the study and recommendations for enhancing the intervention.

## Results

**Fig. 1:**
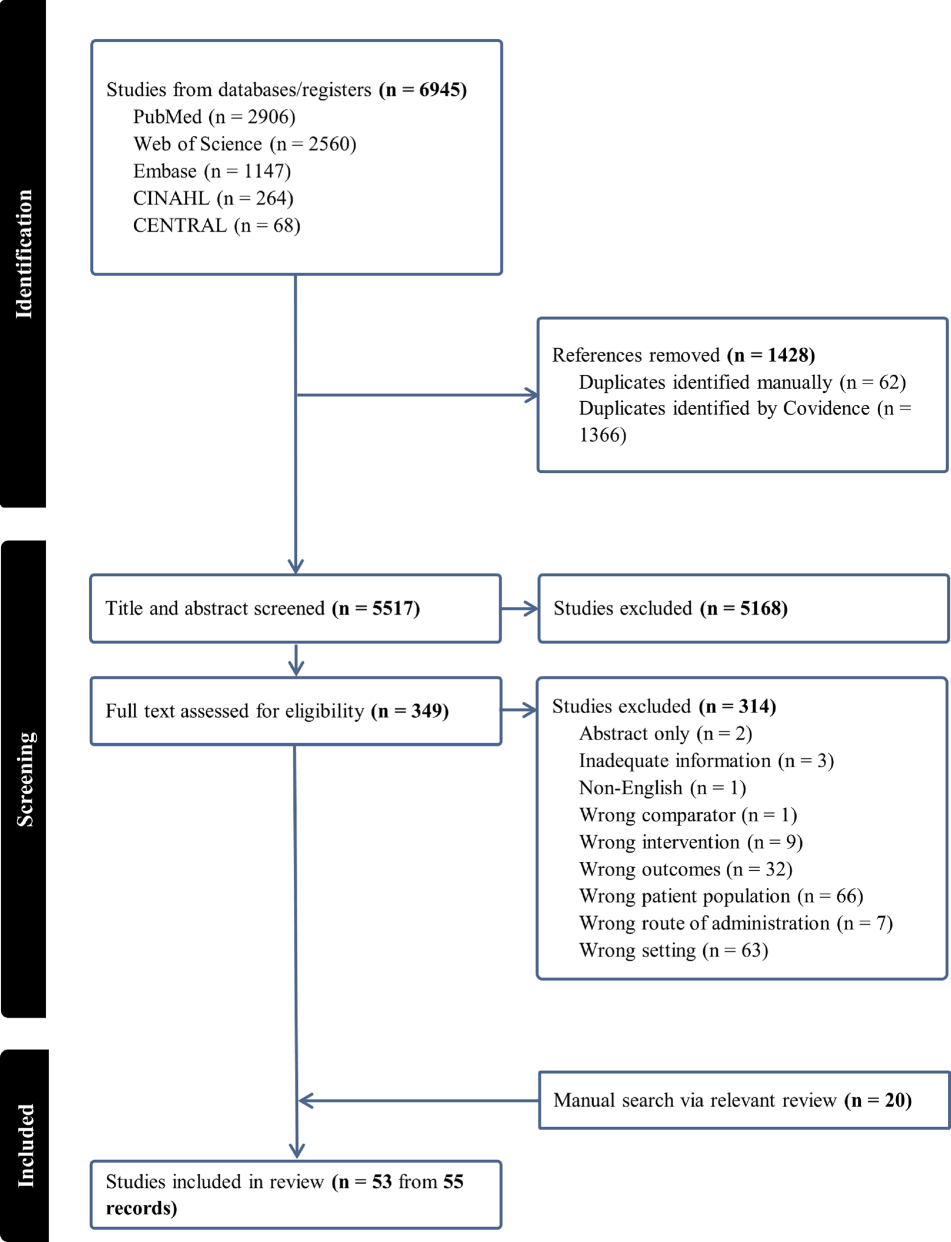
PRISMA scoping review flow chart

Our systematic search yielded a total of 6945 potentially relevant records (Fig. 1). During the initial screening phase, 5168 records were excluded based on title and abstract review. Subsequently, 349 records remained for eligibility assessment. Following a comprehensive screening and selection process, a total of 53 studies from 55 publications met our inclusion criteria and were included in the final review.

### Study design, setting, and population

Table 2 summarizes the characteristics of the included studies. In this scoping review, 24 studies employed an RCT design, 7 studies employed a cluster randomized controlled trial (cRCT) design, 13 studies employed a quasi-experimental design, 4 studies employed a before-after study design, with 1 study being a controlled before-after study, and 4 studies employed a mixed methods design. The included studies were conducted across 13 different countries. The majority of studies were conducted in Asia, comprising 41 studies (77.4%), with the highest number being conducted in Iran (12 studies), followed by China (7 studies), India and Turkey (5 studies each), Malaysia (4 studies), Indonesia and Thailand (3 studies each), and Jordan and Nepal (1 study each). The remaining 9 studies (17%) were conducted in South America, with the majority being conducted in Brazil (8 studies). Lastly, only 2 studies (3.6%) and 1 study (1.8%) were conducted in North America (Mexico) and Africa (South Africa), respectively. Overall, the participants’ ages ranged from 8 to 75 years old, with 23 studies focused on children or adolescents (aged below 18 years), and youth (aged 19–24 years), nine studies targeted adults (aged 25–59 years) to the elderly (aged > 60 years) population, three studies exclusively focused on the elderly aged 60 years and above, and 17 studies targeted the general population. Lastly, only one study did not report its target group. The total number of participants involved in these studies ranged from 27 to 15,310. Additionally, six studies were conducted among pregnant women, and twelve studies were conducted among females only.

**Table 2:** Summary of the included studies

No	Study, year (country)	Study design	Participants’ characteristics	Description of intervention, delivery platform, control, and Theoretical Framework (TF)	Duration, frequency, and follow-up	Summary of results
1	Adam, 2021 (South Africa) [13]	cRCT	Samples: 924 Age: 18 years and above Gender: Females (pregnant)	Intervention: Standard care counselling and mobile video intervention for exclusive breastfeeding including the benefits, recommendations, challenges of breastfeeding, unsafe infant feeding practices, and breastfeeding in HIV Delivery platform: Mobile video Control: Receiving standard care TF: Elaboration likelihood model	Duration: 1 month Frequency: every home visit Follow-up: Post-intervention and 5 months post-intervention	No significant differences in exclusive breastfeeding and complementary feeding practices between the groups at one and 5-month follow-up Significant increase in mother knowledge in the IG at one-month follow-up. However, no significant difference in the knowledge between groups at the 5-month follow-up
2	Alsaleh, 2023 (Jordan) [14]	RCT	Samples: 131 Age: Mean age 21.3 years Gender: Males and females	Intervention: A behavioural PA intervention based on individualized telephone consultation, reminder text messages, and Facebook Delivery platform: SMS and Facebook Control: Received education sessions (lectures) every month for 60 min TF: Social cognitive theory and self-efficacy theory	Duration: 6 months Frequency: Once a month for phone consultation and once a week for SMS Follow-up: Post-intervention	The number of physically active participants had increased significantly in the IG and declined in the CG Moderate PA frequency, duration, and intensity increased significantly in the IG than in the CG
3	Angkasa, 2020 (Indonesia) [15]	Quasi-experimental	Samples: 228 Age: 9–12 years Gender: Males and females	Intervention: Internet-based video game about healthy food and balanced diet in addition to nutrition education using leaflets and poster Delivery platform: Video game Control: Received standard nutrition education (using leaflets and posters) for 10–15 min TF: Not reported	Duration: 8 days Frequency: Two consecutive days, 30 min each day Follow-up: 30 min and 1 week post-intervention	At both follow-ups, the knowledge score in the IG significantly differed compared to the CG The mean changes in attitude score for IG also significantly differed compared to the CG at both follow-ups
4	Bhattarai, 2023 (Nepal) [16]	Mixed methods process evaluation	Samples: 35 Age: 13–49 years Gender: Females (pregnant)	Intervention: Online or virtual counselling intervention to improve diet and iron intake Delivery platform: Zoom and WhatsApp Control: Received routine antenatal care TF: Not reported	Duration: 3 months Frequency: 2 times virtual counselling with 14 days interval Follow-up: Not reported	Acceptability: Most participants were enthusiastic and reported that they learned useful and new information Feasibility: Scheduling was not a problem, but they were often busy with household or farm work. Mostly they asked to complete the counselling quickly Challenges: Poor network, unreliable internet reception, many did not have enough digital literacy, and it was difficult for other family members to participate
5	Bombem, 2013 (Brazil) [17]	cRCT	Samples: 124 Age: 18–64 years Gender: Males and females	Intervention: The messages about healthy diet and PA guidance Delivery platform: E-mails Control: No intervention TF: Intervention mapping framework and cognitive social theory	Duration: 6 months Frequency: Monthly Follow-up: Post-intervention	The intervention had a significant impact on increasing the dietary quality index and four components (grain, vegetables, milk, and dairy products) and a reduction in meat and eggs
6	Da Silva, 2019 (Brazil) [18]	cRCT	Samples: 895 Age: 7th to 9th graders Gender: Males and females	Intervention: StayingFit—online program to encourage and guide weight control and healthy eating habits Delivery platform: Website Control: No intervention TF: Cognitive behavioural theory	Duration: 12 months Frequency: Once a week for 30 min each Follow-up: Post-intervention	No significant difference concerning the anthropometric parameters The chance of consuming beans significantly increased, while the chance of regularly consuming soft drinks reduced
7	Cai, 2022 (China) [19]	cRCT	Samples: 72 Age: ≥ 60 years Gender: Males and females	Intervention: Peer support, face-to-face PA sessions, and a mobile application-based walking program about the benefits of PA and behaviour change techniques Delivery platform: WeChat Control: Received lecture, booklet, and phone call once a month TF: Social cognitive theory and the health action process approach model	Duration: 3 months Frequency: Once a week Follow-up: Post-intervention	PA self-efficacy was significantly improved in the IG than in the CG No significant group difference for any body composition measures between the IG and CG
8	Cai, 2023 (China) [20]	Quasi-experimental	Samples: 444 Age: Mean age 18.4 years Gender: Males and females	Intervention: Food safety-related popular science articles released by WeChat Delivery platform: WeChat Control: No intervention TF: Not reported	Duration: 2 months Frequency: Weekly Follow-up: Post-intervention	After the intervention, compared to the CG, the IG did not score significantly higher on knowledge, attitude, and practice of food safety
9	Ceylan, 2022 (Turkey) [21]	Quasi-experimental	Samples: 185 Age: Mean age 15.6 years Gender: Males and females	Intervention: Text messages contained basic information on PA and encouraging messages on increasing self-efficacy and diminishing barriers Delivery platform: WhatsApp Control: No intervention TF: The transtheoretical model	Duration: 8 weeks Frequency: Once a day Follow-up: 4 weeks post-intervention	The IG had a higher exercising self-efficacy score, MET score for performing vigorous PA, and total MET score compared to the CG No significant difference in BMI
10	Chagas, 2020 (Brazil) [22]	cRCT	Samples: 319 Age: 13–19 years Gender: Males and females	Intervention: Rango cards game about healthy eating practices, food labels, and nutritional content of food items Delivery platform: Video games Control: No intervention TF: Social cognitive theory	Duration: 17 days Frequency: daily Follow-up: Not reported	Significant reductions in the IG compared to the CG for the habit of eating while watching TV or studying and having meals at fast food restaurants Increased knowledge of the effects of fruit and vegetable consumption and improved self-efficacy in the adoption of healthy eating practices in the IG
11	Coknaz, 2019 (Turkey) [23]	Mixed method	Samples: 106 Age: 8–13 years Gender: Males and females	Intervention: Active video games including sports, aerobics, results and training Delivery platform: Video games Control: No intervention TF: Not reported	Duration: 12 weeks Frequency: 3 days a week Follow-up: Post-intervention	At the end of the intervention, BMI and BMI z-score decreased significantly in the IG compared to increased value in the CG No significant effect of the intervention on fat ratio Acceptability: Participants had positive feelings of enjoyment, excitement, and activity and energy. Meanwhile, some stated negative feelings of dislike and boring
12	De Barros, 2009 (Brazil) ‡ [24]	cRCT	Samples: 2155 Age: 15–24 years Gender: Males and females	Intervention: Saúde na Boa—focused on simple environmental/organizational changes, diet and PA education, and personnel training Delivery platform: Multicomponent (lectures, posters, newsletter distribution, and website) Control: No intervention TF: Not reported	Duration: 9 months Frequency: weekly Follow-up: Post-intervention	At the post-intervention phase, the proportion of subjects meeting current PA recommendation was higher in the IG group than in the CG group A reduction in the prevalence of inactivity was observed in the IG group and a rise was found in the CG group
13	Duan, 2017 (China) [25]	RCT	Samples: 132 Age: 17–24 years Gender: Males and females	Intervention: Web-based intervention targeting social-cognitive indicators for health behavioral change for PA and fruit and vegetable intake Delivery platform: Website Control: No intervention TF: Not reported	Duration: 8 weeks Frequency: Once a week Follow-up: Post-intervention and 1-month post-intervention	Inactive individuals in the IG at baseline were more likely to be active at the end of the intervention and at 1-month follow-up Significant changes between IG and CG, both at the end of the intervention and at the 1-month follow-up for previously inactive and active individuals
14	Espinosa-curiel, 2020 (Mexico) [26]	Before-after study	Samples: 60 Age: 8–10 years Gender: Males and females	Intervention: FoodRateMaster—video games education about characteristics of healthy and unhealthy food Delivery platform: Video games Control: None (pre-post design) TF: Behavioral, cognitive, and social cognitive theory	Duration: 45 days Frequency: 12 game sessions, 3.5 h in total Follow-up: Post-intervention (game session)	Participants showed increased food knowledge from pregame to postgame play Greater self-reported frequency in the consumption of cauliflower, broccoli and corn quesadillas Lower self-reported intake of 10 unhealthy foods, including french fries, candy and chocolate, sweet soft cakes, and soft drinks
15	Espinosa-curiel, 2022 (Mexico) [27]	RCT	Samples: 27 Age: 8–11 years Gender: Males and females	Intervention: HelperFriend—video games to increase knowledge of PA, healthy eating, and socio-emotional wellness Delivery platform: Video games Control: Received a 45-min talk about the importance of healthy behaviours TF: Not reported	Duration: 4 weeks Frequency: 6 game sessions, with 30 min each session over 21 days Follow-up: Post-intervention	Significantly increased PA and healthy eating knowledge in the IG than the CG Intention to perform healthy behaviours (PA and healthy eating) in the IG significantly increased than the CG The IG reported reduced consumption of ham, sausage, soft drinks, burritos, hamburgers, salt peanuts, sweet cookies, potato chips, cake, and sweet soft cakes
16	Gadenz, 2024 (Brazil) [28]	RCT	Samples: 147 Age: Mean age 35.2 years Gender: Males and females	Intervention: Dieta Dash application with meal evaluation, healthy meals, healthy choices, and healthy recipe information Delivery platform: Smartphone app Control: Usual care and conventional telehealth support tools TF: Not reported	Duration: 4 weeks Frequency: Able to access the App at any time Follow-up: 30 days post-intervention	No significant differences in the mean DASH diet knowledge between the IG and CG
17	Gutierrez-Martinez, 2018 (Colombia) [29]	RCT	Samples: 120 Age: 5th -grade students Gender: Males and females	Intervention: Active Module of Active Recess (MARA) that includes 30 PA sessions that combine games with elements such as bows, balls, hoops, ladders, rumba, and gaming sessions as well as SMS messages (MARA + SMS) Delivery platform: Face-to-face and SMS Control: No intervention TF: Not reported	Duration: 10 weeks Frequency: 3 times a week Follow-up: Post-intervention	Significant increase in the mean daily minutes of light, moderate, and vigorous PA in the IG in relation to the CG The minutes of sedentary behaviours decreased in the IG and increased in the CG No significant changes in adiposity and BMI between groups at post-intervention
18	Gür, 2020 (Turkey) [30]	RCT	Samples: 128 Age: 18 years and above Gender: Males and females	Intervention: Exercise education component (i.e., goal setting, time management skills, self-efficacy) and exercise prescription Delivery platform: Smartphone application—ERVE Control: No intervention TF: Cognitive behavioural theory	Duration: 8 weeks Frequency: 4 times a week for 30–45 min each time Follow-up: Post-intervention	BMI decreased for the IG while increased in the CG PA increased in the IG, while inclined in the CG
19	Hanafiah, 2022 (Malaysia) [31]	RCT	Samples: 305 Age: 20–39 years Gender: Females	Intervention: The Jom Mama—app provides information on healthy lifestyles including healthy food and PA challenges Delivery platform: Counselling and smartphone app Control: Receiving standard care TF: Not reported	Duration: 33 weeks Frequency: Once in 6 contact points Follow-up: Post-intervention	The IG took less of rice as compared to the CG Significant increase in moderate to vigorous PA in the IG compared to the CG No significant reduction in WC between groups BMI increased in both groups
20	Hardman, 2014 (Brazil) ‡ [32]	cRCT	Samples: 989 Age: 15–24 years Gender: Males and females	Intervention: School-based intervention program that includes culturally relevant activities, physical education, and health promotion Delivery platform: Website Control: No intervention TF: Not reported	Duration: 9 months Frequency: Weekly Follow-up: Post-intervention	The intervention had no significant effect on reducing exposure to screen time
21	He, 2017 (China) [33]	Quasi-experimental	Samples: 15,310 Age: 18 years or older Gender: Males and females	Intervention: WeChat-based weight loss intervention program Delivery platform: WeChat Control: Receiving routine publicity on weight loss TF: Not reported	Duration: 6 months Frequency: At any time Follow-up: Post-intervention	For males, the IG had a higher probability of maintaining weight, weight loss from 1 to 2 kg, or weight loss more than 2 kg than the CG No significant difference between maintaining weight and weight gain for females in both groups The more active participants engaged in the weight loss program, the more weight they lost
22	Jorvan, 2020 (Iran) [34]	Quasi-experimental	Samples: 114 Age: Mean age 37.6 years Gender: Males and females	Intervention: Health belief model-based education and exercise intervention Delivery platform: Telegram messenger Control: No intervention TF: Health belief model	Duration: 2 weeks Frequency: twice within 2 weeks Follow-up: 6 months post-intervention	Significant difference in relation to daily and weekly exercises and self-perceived exercise in the IG, but not in the CG
23	Kato-Lin, 2020 (India) [35]	RCT	Samples: 102 Age: 10–11 years Gender: Males and females	Intervention: Fooya!—a mobile game about healthy eating Delivery platform: Video games Control: Playing board game which does not deliver any knowledge about healthy eating TF: Mechanics, dynamics, aesthetics (MDA) framework	Duration: 2 days Frequency: 20 min per day Follow-up: 1 week post-intervention	Significantly higher main effect of the mobile game on the number of healthy foods actually chosen and identified in the IG than the CG
24	Kaur, 2023 (India) [36]	Mixed method process evaluation	Samples: 668 Age: 35–70 years Gender: Males and females	Intervention: “SMART Eating”—nutrition education intervention Delivery platform: Website, SMS, and WhatsApp Control: Receiving traditional nutrition education through pamphlets on dietary recommendations for Indian TF: UK Medical Research Council’s (MRC) framework	Duration: 6 months Frequency: Weekly Follow-up: Post-intervention	Significant increase of fat, sugar, salt (FSS), and fruit and vegetable (FV) intake in the IG compared with the CG Monthly household purchases of FSS reduced significantly in the IG, while it remained constant overtime in the CG FV purchase increased significantly in the IG, while it reduced significantly in the CG Acceptability: The intervention was useful and well-liked. Some expressed interest in extending the program Feasibility: Access to the website (92%), access to SMS (100%), access to WhatsApp (91%) Challenges: lack of family support, tempting tastes, and high cost and poor quality of healthy food as the main reasons for no perceived effect of the intervention
25	Khayat, 2022 (Iran) [37]	Quasi-experimental	Samples: 120 Age: 10–19 years Gender: Females (pregnant)	Intervention: Telemedicine and face-to-face training on adolescent pregnant women about personal hygiene, nutrition, exercise, smoking cessation, pregnancy care, and pregnancy risk Delivery platform: WhatsApp Control: No intervention TF: Self-efficacy theory	Duration: Not reported Frequency: Every 2 days Follow-up: Every week until the end of the 28th week of pregnancy	Significant improvement in scores of exercise and PA in the IG compared to the CG
26	Khorshid, 2014 (Iran) [38]	RCT	Samples: 93 Age: Mean age 25.5 years Gender: Females (pregnant)	Intervention: Reminders and educational health messages about iron supplementation Delivery platform: SMS text messages Control: Usual health care TF: Not reported	Duration: 12 weeks Frequency: Once a week Follow-up: Post-intervention	No significant difference between haemoglobin, hematocrit, and ferritin levels between IG and CG Women in the IG had higher compliance with iron supplements compared to the CG
27	Kiani, 2021 (Iran) [39]	Quasi-experimental	Samples: 93 Age: Mean age 25.1 years Gender: Females (pregnant)	Intervention: Mobile-application intervention which contained education such as PA during pregnancy, different types of pregnancy exercise, planning for exercise, demonstration, and educational videos Delivery platform: Mobile application Control: Received childbirth preparation classes TF: Not reported	Duration: 3 months Frequency: During the week Follow-up: Post-intervention	The total mean score of PA significantly increased in the IG, while the change decreased in the CG Significant difference in the mean score of PA between groups after intervention
28	Leme, 2016 (Brazil) [40]	RCT	Samples: 253 Age: 14–18 years Gender: Females	Intervention: Healthy Habits, Healthy Girls (H3G) program based on ten nutrition and PA messages to support healthy eating and regular PA Delivery platform: Handbooks, seminars, workshops, newsletter, health messages via WhatsApp, and diary Control: No intervention, but received a condensed version of the program after follow-up assessment TF: Social cognitive theory (SCT)	Duration: 6 months Frequency: Weekly Follow-up: Post-intervention	Significant intervention effects between groups for waist circumference, computer screen time on the weekends, total sedentary activities on the weekends, and fruit and vegetable intake No significant between-group differences in BMI and BMI Z-score
29	Liu, 2024 (China) [41]	RCT	Samples: 153 Age: 18 years and above Gender: Males and females	Intervention: Applet-based personalized dietary intervention on dietary intake Delivery platform: Social media via WeChat and mobile app (Applet) Control: No intervention TF: Not reported	Duration: 4 months Frequency: every weekday Follow-up: Post-intervention	No significant differences between groups in relation to body weight, BMI, body composition, percentage of body fats, and visceral fat index at the end of the study Significant weekly decrease in the animal/plant food ratio and the intake of livestock and poultry meat in the IG compared with the CG No significant increase between groups in the intakes of vegetables and fruits and plant-based foods
30	Lua, 2013 (Malaysia)^†^ [42]	cRCT	Samples: 380 Age: 18–24 years Gender: Males and females	Intervention: Nutrition education intervention related to healthy food, recommended intake, maintaining body weight, PA, food labels, and food preparation Delivery platform: Lecture, brochure, and text messaging Control: No intervention TF: Not reported	Duration: 10 weeks Frequency: Every five days Follow-up: Post-intervention	No significant differences between groups in relation to body weight, BMI, waist circumference, and hip circumference at post-intervention Significant decrease in sitting in the IG than the CG at post-intervention Significant increase in weekly moderate and vigorous activity in the IG compared to the CG at post-intervention
31	Maden, 2022 (Turkey) [43]	RCT	Samples: 44 Age: 18–28 years Gender: Males and females	Intervention: Virtual reality training (VRT) and aerobic training (AT) intervention Delivery platform: Video games (Exergame) Control: No intervention TF: Not reported	Duration: 6 weeks Frequency: 3 days a week, with 30 min each Follow-up: Post-intervention	Post-training, the IGs had significantly shorter gaming time and sedentary time compared to the CG Post-training, weekly PA increased significantly in the IGs compared to the CG
32	Mehran, 2012 (Iran) [44]	RCT	Samples: 205 Age: 18 years and above Gender: Females	Intervention: The text messages consisted of information on the definition and consequences of iodine deficiency and the importance of consumption and proper storage of iodised salt Delivery platform: SMS Control: Received brief information on iodised salt via fixed-line telephone TF: Not reported	Duration: 6 weeks Frequency: Daily Follow-up: Week 8 (2 weeks post-intervention)	Significant difference in knowledge and attitude scores between the IG and CG at follow-up No significant differences between groups in behaviour as reflected by practice scores and salt and urinary iodine levels
33	Müller, 2016 (Malaysia) [45]	Mixed methods	Samples: 43 Age: 55–70 years Gender: Males and females	Intervention: SMS texting intervention on PA, including the benefits of PA and safety instructions Delivery platform: Booklet and SMS Control: Received no SMS TF: Not reported	Duration: 12 weeks Frequency: 5 SMS weekly Follow-up: Post-intervention and at week 24	No significant differences between groups on BMI, weekly exercise frequency, and exercise self-efficacy at weeks 12 and 24 Acceptability: Participants affirmed the value of the SMS text as helpful and inspiring Challenges: Personal barriers such as laziness, tiredness, and lack of motivation in the IG
34	Nurgul, 2015 (Turkey) [46]	Before-After study	Samples: 30 Age: 18–55 years Gender: Females	Intervention: Web-based health promotion consisted of three modules, including nutrition elements, cooking and preserving, and PA Delivery platform: Website Control: None (pre-post design) TF: Not reported	Duration: 3 months Frequency: Able to access at any time Follow-up: Post-intervention	Significant difference in the knowledge of PA and nutrition before and after training
35	Panahi, 2021 (Iran) [47]	Quasi-experimental	Samples: 140 Age: 18–65 years Gender: Males and females	Intervention: Health belief model-based intervention with content related to a healthy lifestyle to prevent osteoporosis, the role of nutrition, dietary benefits and barriers, dietary recommendations, and the role of exercise Delivery platform: Social media (Telegram or WhatsApp) Control: No intervention TF: Health belief model	Duration: 4 weeks Frequency: Once a week Follow-up: 3 months post-intervention	Significant difference between groups in the mean scores of nutrition behaviours and walking behaviours at three months post-intervention Significant difference between mean scores of nutrition and walking behaviour in the IG before and after intervention, while no significant difference was observed in the CG
36	Pentakota, 2019 (India) [48]	Before-After study	Samples: 350 Age: Mean age 18.9 years Gender: Males and females	Intervention: Health fitness app (Runtastic) which had the facility to send reminder notifications for PA, display achievements in terms of calories burnt, minutes spent in PA, and kilometres Delivery platform: Mobile app Control: None (pre-post design) TF: Not reported	Duration: 1 month Frequency: Not reported Follow-up: Post-intervention	Significant increase in the leisure time PA and decrease in BMI after 1-month intervention
37	Peyman, 2018 (Iran) [49]	Quasi-experimental	Samples: 360 Age: 18 years and above Gender: Females	Intervention: Digital media-based health intervention including educational websites and films related to PA and SMS messages about the importance of PA Delivery platform: Website, CD, and SMS Control: No intervention TF: Not reported	Duration: 6 months Frequency: Website and educational films can be accessed every time, SMS sent daily Follow-up: Post-intervention	Significant difference before and after the intervention concerning the mean score of knowledge, attitude, and level of PA in the IG, but not in the CG The difference between the two groups in terms of knowledge, attitude, and PA was significant after the intervention
38	Pfammatter, 2016 (India) [50]	RCT	Samples: 1925 Age: 18 years and above Gender: Males and females	Intervention: mDiabetes, a text messaging program to improve diabetes risk behaviours and increase awareness about the cause and complications of diabetes Delivery platform: SMS Control: No intervention TF: Not reported	Duration: 6 months Frequency: Daily for the first 6 days, then twice a week Follow-up: Post-intervention	The IG demonstrated greater improvement in a health behaviour composite score compared to the CG Improved fruit, vegetables, and fat consumption but not PA were observed in the IG as compared with CG at the end of the study
39	Pomkai, 2024 (Thailand) [51]	RCT	Samples: 80 Age: 20–40 years Gender: Males and females	Intervention: The light-hearted app, a mobile-based app education to increase awareness of PA and motivation to do PA Delivery platform: Mobile app and Line Control: No intervention TF: Not reported	Duration: 8 weeks Frequency: Not reported Follow-up: Post-intervention	Participants in the IG had higher weekly moderate-vigorous PA than those in the CG
40	Rachmah, 2023 (Indonesia) [52]	Before-After study	Samples: 200 Age: 18 years and above Gender: Females	Intervention: Ten nutritional education sessions on complementary feeding Delivery platform: WhatsApp with PowerPoint presentation, posters, and video tutorials Control: None (pre-post design) TF: Theory of planned behaviour construct	Duration: 8 days Frequency: daily, each session 4 h Follow-up: Post-intervention	The intervention increased the mother’s knowledge, attitude, subjective norm, perceived behavioural control, self-efficacy, and intention toward giving nutritious complementary feeding
41	Rerksuppaphol, 2017 (Thailand) [53]	RCT	Samples: 217 Age: Mean age 10.7 years Gender: Males and females	Intervention: Internet-based obesity prevention program including information on healthy nutrition, food habits, and PA Delivery platform: Website Control: Receiving informed knowledge of proper healthy diet, PA, and sedentary behaviours TF: Not reported	Duration: 4 months Frequency: Not reported Follow-up: Post-intervention	Significantly higher BMI, BMI z-score, and waist-to-height ratio in the CG than in the IG at the end of the study Compared to their baseline, no changes in weight z-score and BMI z-score in the IG at the end of the study Compared to their baseline, significant increases in weight z-score and BMI z-score in the CG at the end of the study
42	Rica, 2020 (Brazil) [54]	RCT	Samples: 50 Age: 60 years and above Gender: Females	Intervention: Kinect-based training program (balance games, strengths, and light aerobic tasks) Delivery platform: Video games Control: Playing board games and continue normal daily activities TF: Not reported	Duration: 12 weeks Frequency: 3 times a week with 60 min per session Follow-up: Post-intervention	No changes in anthropometric and body composition in both groups after the intervention
43	Sabooteh, 2021 (Iran) [55]	Quasi-experimental	Samples: 206 Age: Not reported (university students) Gender: Males and females	Intervention: “Health to infinity”—an educational program targeting PA behaviors (software group and web group) Delivery platform: Software, Website, SMS, and email Control: No intervention TF: Pender Health Promotion Model	Duration: One month Frequency: Daily Follow-up: Post-intervention, two, and six months post-intervention	Significantly higher in the mean score of PA in the IGs compared to the CG at all follow-up times No significant differences concerning the mean score of knowledge between the IGs and CG before the intervention as well as at all follow-up times Significant difference in the mean score of attitude at all follow-up times between the IGs and CG
44	Santana, 2024 (Brazil) [56]	RCT	Samples: 86 Age: 18–59 years Gender: Males and females	Intervention: Online nutritional (teleconsultation) counselling program (traditional group) or along with an educational booklet explaining aspects related to healthy eating, healthy recipes, messages, and nutrition education actions (multicomponent group) Delivery platform: Virtual meetings and emails Control: Receiving a single educational booklet TF: Not reported	Duration: 12 weeks Frequency: Three times teleconsultation, weekly messages Follow-up: Post-intervention	Significant increase in eating practice score both in the IGs compared to the CG at the end of the intervention
45	Sari, 2022 (Indonesia) [57]	Quasi-experimental	Samples: 277 Age: 15–18 years Gender: Females	Intervention: m-Health using WANTER application—which contains information related to iron deficiency anaemia, BMI, balanced nutrition, and a consultation menu regarding anaemia Delivery platform: Mobile app Control: Receiving booklet TF: Not reported	Duration: 3 months Frequency: Not reported Follow-up: Post-intervention	No significant difference between the IG and CG on knowledge, attitudes, and practices related to anaemia, either before or after treatment
46	Seyyedi, 2020 (Iran) [58]	RCT	Samples: 100 Age: Mean age 30.0 years Gender: Females	Intervention: Maternal education for complementary feeding, including nutrition principles based on child age, method for child feeding, and mothers’ health Delivery platform: Mobile app and email Control: Receiving usual treatment TF: Not reported	Duration: 6 months Frequency: At any time, a reminder email message once a week Follow-up:	A significantly greater improvement concerning the nutritional literacy score, knowledge, attitudes, and practice toward feeding in the IG compared to the CG Children of mothers in the IG showed significantly greater WHZ, WAZ, and HAZ scores than the CG
47	Shahril, 2013 (Malaysia) † [59]	cRCT	Samples: 380 Age: 18–24 years Gender: Males and females	Intervention: Nutrition education intervention which comprised three themes such as always being healthy (variety of food, body weight, and PA), eating moderately (portion size), and living the future (information related to food to avoid) Delivery platform: Lectures, brochures, and SMS Control: No intervention TF: Not reported	Duration: 10 weeks Frequency: Text message sent once every 5 days Follow-up: Post-intervention	Better intakes of calcium, vitamin C, and thiamine in the IG compared to the CG Significantly increased intakes of fruits and 100% fruit juice, fish, egg, milk, and dairy products in the IG compared to the CG after 10 weeks Intake of processed foods decreased significantly over 10 weeks in the IG compared to the CG
48	Sharma, 2017 (India) [60]	CBA	Samples: 382 Age: 18–64 years Gender: Males and females	Intervention: mHealth intervention on modifying behavioural risk factors of non-communicable diseases Delivery platform: Phone call and SMS Control: No intervention TF: Not reported	Duration: 8 months Frequency: Text message sent once a week, once a month for a phone call Follow-up: 2 months post-intervention (at month 10)	The mean number of daily servings of fruits and vegetables increased in the IG and the CG at follow-up Significantly increased PA in the IG and no significant change in the CG Significant reduction of BMI in the IG, while a significant increase of BMI in the CG
49	Shukri, 2019 (Malaysia) [61]	Quasi-experimental	Samples: 201 Age: Mean age 10.5 years Gender: Males and females	Intervention: Computer-based teaching method to support healthy eating Delivery platform: Computer-based games and animated presentation Control: No intervention TF: Not reported	Duration: 3 weeks Frequency: 30 min per gameplay, 4.5 h in total Follow-up: Post-intervention and after 12 months	No significance of dietary knowledge score between pre-and post-test in both groups No significant changes in intention to eat healthy or limit unhealthy food, between pre-and post-test in both groups At the post-test, the IG had a significantly higher intention to limit unhealthy eating and a more negative attitude towards unhealthy food than the CG No changes in attitudes towards healthy food between pre-and post-test in both groups At the post-test, a significant increase in vegetable consumption and a decrease in fast food, soft drink, junk food, and sweet food consumption in the IG compared to the CG At the 12-month follow-up, a significant reduction in fruit and vegetable consumption and an increase in junk food and sweet food consumption among the IG
50	Sriramatr, 2014 (Thailand) [62]	RCT	Samples: 220 Age: 18–24 years Gender: Females	Intervention: Internet-based intervention for promoting and maintaining PA Delivery platform: Website and email Control: No intervention TF: Social cognitive theory	Duration: 3 months Frequency: once a week Follow-up: Post-intervention and 3 months post-intervention (at month 6)	A significantly higher leisure time activity score (LTAS) in the IG than in the CG at the end of the intervention and follow-up A significantly higher self-efficacy of PA in the IG than in the CG at the end of the intervention and follow-up
51	Talebi, 2022 (Iran) [63]	RCT	Samples: 71 Age: Mean age 28–29 years Gender: Females (pregnant)	Intervention: Social media education with special and prepared educational content (written, audio, and video) to improve PA, individually designed diet, and reminder messages Delivery platform: WhatsApp Control: Receiving routine pregnancy care, prepared individual diets, and materials on weight gain during pregnancy TF: Not reported	Duration: 8 weeks Frequency: twice a week Follow-up: Post-intervention	No significant change in mean weight and sedentary activities between groups after the intervention Significantly higher total daily, light, moderate, and severe PA in the IG than CG after the intervention
52	Vahedian-Shahroodi, 2021 (Iran) [64]	RCT	Samples: 202 Age: 18–50 years Gender: Females	Intervention: Theory-based educational intervention for enhancing nutrition, healthier diet, food selections and promoting healthier active lifestyles, which includes nine training sessions and online counselling Delivery platform: Multicomponent (lecture, slides, teach-back video, pamphlets, focus group discussion, phone contact, and Telegram) Control: No intervention TF: Health promotion model	Duration: 3 months Frequency: 4 h training sessions (every 10 days) Follow-up: Post-intervention and 3 months post-intervention (month 6)	Significant difference in the change PA behaviours (prior behaviour, self-efficacy, activity-related affect, perceived barriers or benefits, commitment, and behaviour outcome) in the IG compared with the CG at follow-up Significant improvement in nutrition behaviours (prior behaviours, self-efficacy, perceived barriers or benefits, commitment, and behaviour outcome) in the IG vs. CG at follow-up, and in change from baseline to follow-up
53	Vakili, 2015 (Iran) [65]	RCT	Samples: 92 Age: 40–60 years Gender: Females	Intervention: Short messaging to enhance and motivate the consumption of healthy food Delivery platform: SMS Control: No intervention TF: Not reported	Duration: 4 months Frequency: once a week Follow-up: Post-intervention	The consumption of vitamin A-rich fruit, vegetables, and fish increased significantly in the IG compared to the CG after intervention No significant increase in the consumption of green leafy vegetables and dairy products between groups
54	Wang, 2022 (China) [66]	RCT	Samples: 201 Age: 65–75 years Gender: Males and females	Intervention: Internet-based nutrition and exercise interventions on the prevention and treatment of sarcopenia which include dietary management information (nutrition group), exercise management information (exercise group), or both (comprehensive group) Delivery platform: Mobile App Control: Receiving health education without any other intervention TF: Not reported	Duration: 12 weeks Frequency: Once in 2 weeks Follow-up: Post-intervention	No significant differences between groups in energy intake, protein intake, and moderate PA Significant differences among the groups in the daily average intake of high-quality protein and BMI Significant changes in skeletal muscle mass among the groups before and after the intervention
55	Zhou, 2021 (China) [67]	Quasi-experimental	Samples: 704 Age: 9–11 years Gender: Males and females	Intervention: Classroom-based Brain Breaks intervention on PA attitude including information about the importance of exercise habits, self-efficacy, exercise motivation and enjoyment, and self-confidence Delivery platform: Video Control: No intervention TF: Not reported	Duration: 3 months Frequency: Daily with an accumulated time of 30 min Follow-up: Post-intervention	No effect of classroom video interventions on children’s self-efficacy to do PA

^†,‡^Record from the same study

*BMI* body mass index, *CG* control group, *cRCT* cluster randomized controlled trial, *IG* intervention group, *PA* physical activity, *RCT* randomized controlled trial

### Intervention characteristics

**Fig. 2:**
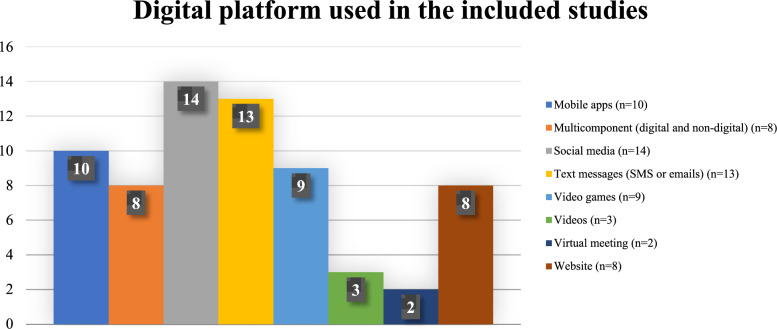
Digital platforms listed in the included studies

The duration of interventions varied considerably, spanning from as short as 2 days to as long as 12 months. The majority of studies (31 studies) implemented interventions lasting one to three months, followed by 11 studies with intervention durations of four to six months. Short-term interventions lasting less than a month were implemented in six studies, and four studies extended their interventions above six months. Notably, one study did not provide information on the length of its intervention. Across 53 studies, social media and text messages via SMS or emails were widely used for the delivery platform (21% and 19% respectively), followed by mobile apps (15%), video games (13%), and both websites and multicomponent platforms—which utilized a combination of digital and traditional methods—each accounting for 12% of the interventions (Fig. 2). In this review, 'multicomponent interventions' refer to interventions that combine two or more delivery platforms or strategies, which typically involve a combination of digital platforms and traditional methods (such as in-person counselling sessions, printed educational materials, or workshops).

Notably, studies targeting adolescents and young adults mostly utilized video games as their platform, which was reported in eight studies. This was followed by multicomponent interventions, social media, and websites, each utilized in four studies. Mobile apps and text messages (SMS and emails) accounted for two studies, and lastly, video was used in only one study in this population (Table 3). In contrast, studies targeting adults and the elderly mostly utilized social media, mobile apps, and text messages, which were reported in four studies each. Lastly, studies among the general population mostly used social media and text messages as their digital intervention platform.

**Table 3:** Distribution of digital platform by age group of the participants

Age group	Total publication	Platform							
		Mobile apps	Multicomponent	Social Media	Text messages (SMS or emails)	Video games	Videos	Virtual Meeting	Website
Children, adolescents, and youth	25	[48, 57]	[24, 32]^‡^ [29, 40] [42, 59]^†^	[14, 20, 21, 37]	[14, 62]	[15, 22, 23, 26, 27, 35, 43, 61]	[67]	–	[18, 25] [53, 62]
Adults and elderly	12	[28, 39, 58, 66]	[45]	[19, 34, 36, 63]	[36, 38, 58, 65]	[54]	–	–	[36]
General population	17	[30, 41, 51]	[31, 64]	[16, 33, 41, 47, 51, 52]	[17, 44, 49, 50, 56, 60]	–	[13, 49]	[16, 56]	[46, 49]
Not specified	1	[55]	–	–	[55]	–	–	–	[55]

^†,‡^Record from the same study

Nineteen studies reported that they developed their intervention based on theoretical frameworks. Specifically, the majority of studies applied social cognitive theory [14, 17, 19, 22, 26, 40, 62], followed by cognitive behavioural theory [18, 26, 30], self-efficacy theory [14, 37], healthy belief model [34, 47], and health promotion model [55, 64]. One study for each utilised the elaboration likelihood model [13], Intervention Mapping Framework [17], health action process approach model [19], transtheoretical model [21], theory of planned behaviour construct [52], Medical Research Council’s (MRC) framework [36], and mechanics, dynamics, and aesthetics (MDA) framework [35]. Nearly a third of the studies (19 out of 53) focused their intervention on PA behaviours, including PA benefits, self-efficacy, barriers, and behaviour change techniques. Seven studies focused their intervention on nutrition-related topics, including healthy food, healthy eating practices, balanced diets, food labels, and nutritional content. Fifteen studies focused on intervention contents that combined nutrition-related topics concerning healthy diets and PA. Maintaining weight and healthy eating habits were reported in only two studies, while contents related to healthy diets, iron, anaemia, and micronutrients were reported in four studies. Additionally, three studies focused their intervention contents on exclusive breastfeeding and complementary feeding education, while only two studies focused on NCD awareness. Lastly, one study solely focused on food safety topics.

### Main findings of the interventions

Table 4 summarizes the effects of the digital platform intervention based on the outcomes of the studies. Sixteen studies assessed PA status, including light, moderate, or vigorous PA, with 13 studies reporting a significant improvement in PA. One study reported inconsistent results, with a statistically significant difference observed only for vigorous PA but not moderate PA between the intervention and control groups [21]. Meanwhile, two studies conducted in India [50] and China [66] utilized SMS and mobile applications found no significant differences in overall PA and moderate PA among groups, respectively.

**Table 4:**
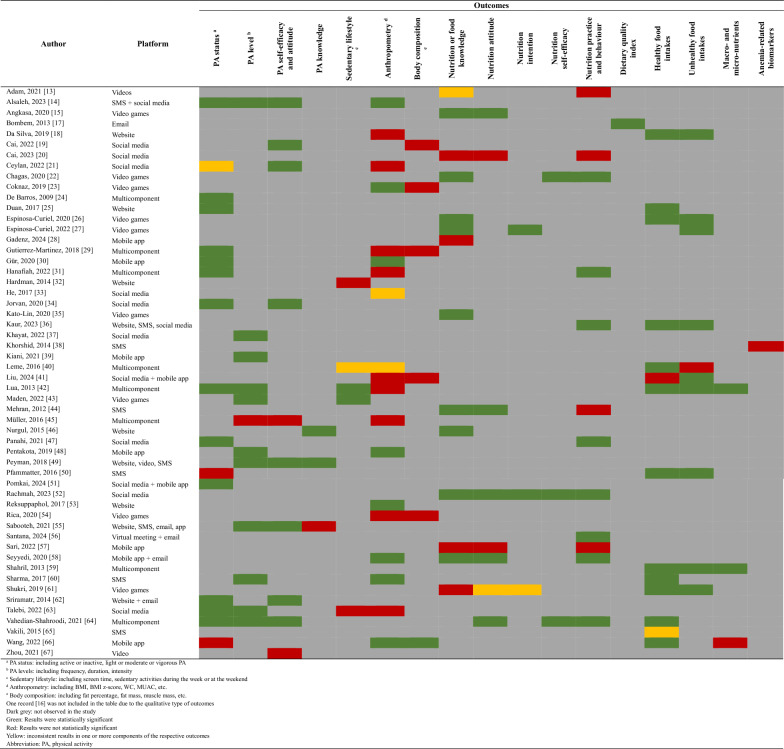
Summary of intervention outcomes

PA level, including frequency, duration, and intensity, was reported in 12 studies, with only one study utilising multicomponent platforms failing to demonstrate significant improvement in PA level [45]. Self-efficacy and attitude toward doing PA were conducted in 10 studies, with two studies conducted among the middle-aged and elderly [45] and adolescents [67] using multicomponent and video interventions, respectively, revealing insignificant improvements in the intervention group. In contrast, only three studies measured PA knowledge as an outcome, with two studies [46, 50] showing a significant improvement in knowledge, while another study that applied four different digital interventions failed to observe a significant improvement in PA knowledge [55].

Sedentary lifestyle outcomes, including screen time and sedentary activities on weekdays and weekends, were assessed in five studies, with only two reporting significant improvements in sedentary behaviours. A study among youth [42] using a multicomponent intervention reported a significant decrease in sitting time. Similarly, a study in Turkey targeting youth reported a significant decrease in sitting time in the intervention group [43]. However, two studies that used multicomponent [32] and social media [63] Interventions targeting adolescents and young adults, respectively, failed to demonstrate a reduction in screen time and sedentary activity. Notably, one school-based study using a multicomponent intervention targeting adolescents in Brazil only showed significant improvement in computer screen time and total sedentary activities on the weekends, but not during the week [40].

A total of 20 studies evaluated anthropometry and body composition as outcome measures, which reported diverse results. Three studies were conducted among adolescents [29], the general population [41], and elderly individuals [54], which utilized multicomponent intervention, social media and mobile applications, and video games, respectively, found no significant improvement in either anthropometric (BMI) or body composition outcomes (including the percentage of body fat, fat mass, and lean mass). Furthermore, a study that employed video games targeting adolescents reported mixed results, with significant improvement in BMI and BMI z-score, but no improvement in percentage fat ratio [23]. In contrast, one study conducted in China among elderly people found improvement in both BMI and body composition, as measured by skeletal muscle mass [66].

A total of nineteen studies assessed knowledge, attitude, intention, self-efficacy, and practice or behaviours (KAISP) related to nutrition. Most studies reported significant improvement in these outcomes after intervention, with only five studies reporting insignificant improvement. Most studies that utilized video games as their digital platform and targeted children, adolescents, and youth found improvement in nutrition-related knowledge outcomes, including balanced and healthy diets, food labels, and nutritional contents [15, 22, 26, 27, 35]. Some of these studies also reported a significant improvement in attitude toward healthy food [15], self-efficacy in the adoption of healthy eating practices [22], and intention and practice towards healthy eating habits (limit fast food) [22, 27]. However, one study in Malaysia that similarly utilized video games as an intervention platform reported no significant improvement in dietary knowledge scores related to healthy eating, intention to eat healthy or limit unhealthy food, and attitudes towards healthy food or unhealthy food [61]. Furthermore, two studies that employed social media [20] and mobile apps [57] platforms among adolescents and youth failed to demonstrate an improvement in knowledge, attitude, and practice in relation to food safety and iron deficiency anaemia, respectively.

Notably, one study conducted among mothers and utilised social media platforms reported significant improvement in all five domains, including knowledge, attitude, intention, self-efficacy, and perceived behavioural control toward giving nutritious complementary feeding to their children [52]. Additionally, a study among mothers who used mobile apps and emails also found significant improvement in knowledge, attitude, and practice toward complementary feeding [58]. Only one study employed a multicomponent platform that assessed the KAISP domain and reported an improvement in attitude, self-efficacy, and behaviour concerning a healthy diet [64].

A total of 23 studies evaluated outcome-related intake, including dietary quality index, consumption of healthy and unhealthy foods, and macro- and micronutrient intake. One study that implemented a digital intervention using e-mails reported significant improvement in the dietary quality index, with an increase in four components of healthy food, including grain, milk, and dairy products [17]. Additional studies focusing on adolescents and youth, which implemented websites [18], video games [26, 61], and multicomponent intervention platforms [59], reported significant improvements in the consumption of healthy food (legumes, dark green vegetables, fruits, fish, egg, and dairy products) and micronutrient intake, including enhanced consumption of calcium, vitamin C, and thiamine. These studies also demonstrated a significant reduction in the intake of less nutritious foods, including sugar-sweetened beverages, fast food, confectionery, potato chips, and processed food, in the intervention group following the intervention period. Notably, studies that implemented a combination of website, social media, and SMS platforms for a six-month intervention period reported improvement in both intakes of more nutritious and less nutritious food [36]. Lastly, other studies conducted in Brazil and China that employed multicomponent interventions [40] or social media and mobile applications [41] reported mixed findings, demonstrating either significant improvement in healthy food intake but insignificant improvement in unhealthy food intake, or vice versa. Finally, our review only recorded a single study that evaluated anaemia-related outcomes [38]. This study implemented SMS platforms to set reminders and deliver educational messages and found no significant difference in the anaemia biomarkers, including haemoglobin, hematocrit and ferritin levels.

## Discussion

Our review explored evidence on digital interventions designed to improve nutritional status and PA in LMICs. This review summarized 55 reports from 53 studies, which covered a range of platform approaches, including mobile apps, social media, text messaging (SMS or emails), video games, video teaching, virtual meetings, and multicomponent approaches that utilized digital and non-digital approaches. These interventions predominantly concentrated on PA and nutrition-related knowledge, attitudes, and practices (KAP), with limited emphasis on topics such as anaemia, micronutrients, feeding practices, NCDs, and food safety. Demographically, most of the studies targeted children, adolescents, and youth (CAY), with only 12 studies targeting exclusively adults and the elderly. Additionally, the majority of studies were conducted in Asia, indicating a gap in interventions in other LMIC regions.

Yet, study on digital health tends to focus on a small number of social inequality indicators and are predominantly conducted in high-income countries, resulting in a gap in understanding the global impact of social inequalities on digital health interventions [68]. With limited cross-regional studies, an opportunity to conduct and expand studies with diverse nutritional contexts and challenges may yield valuable insights into region-specific nutrition and PA problems and how to effectively address them. Such studies could inform the development of more behaviour-specific and sensitive interventions, which are culturally appropriate and tailored to the needs of the target population [69]. Importantly, the effective adoption of digital nutrition interventions relies on the commitment of policymakers and stakeholders to regulation, standardization, and the support of successful implementation and sustainability of these interventions [70]. Policymakers can play a pivotal role by integrating digital health strategies into national health agendas, ensuring adequate infrastructure, fostering public–private partnerships to expand access to digital technologies, and creating an enabling environment for digital health initiatives [71–73]. Furthermore, policy frameworks should address issues of digital literacy, data privacy, and equitable access to ensure that digital interventions reach and benefit all segments of the population [74]. Therefore, it is essential to identify and address specific organizational barriers and facilitators for the effective implementation of digital technologies in community settings within LMICs [74].

Digital education presents a significant opportunity for the design and development of healthcare interventions aimed at disease prevention and self-management. This form of education, which has the potential to enhance health outcomes, is relatively cost-effective, flexible, dynamic, and individually tailored, and can be easily disseminated across large geographical regions. As a result, it has the potential to reach a broad audience, especially those from lower socio-economic backgrounds [75]. Furthermore, certain limitations associated with non-digital intervention programs, such as low motivation, time constraints, and high costs, can be mitigated through the use of digital technology, positioning these digital interventions as viable alternatives to traditional face-to-face methods and facilitating the connection of individual-level interventions with broader socioecological systems [76].

This review also revealed that the majority of the studies did not employ theoretical frameworks in the development of their interventions. The analysis also indicated that research employing theoretical frameworks had a more pronounced impact on the results. The incorporation of theory-based interventions is a significant factor associated with the success of an intervention [14]. Theoretical frameworks guide the intervention by addressing determinants of health behaviour. Theoretical frameworks play a crucial role in enhancing the design of evidence-based interventions, facilitating outcome evaluation, and providing structured approaches for tackling complex health challenges [77]. Frameworks such as intervention mapping facilitate the systematic planning, implementation, and evaluation of health interventions by integrating empirical and theoretical evidence, supporting a comprehensive needs assessment, and enhancing stakeholder engagement [78, 79]. Furthermore, none of the studies reported the implementation of needs assessment and human-centred design (HCD) approaches, which underscores the importance of the target participants and stakeholders' involvement in co-designing the intervention. Applying these approaches in designing health interventions offers numerous benefits that enhance the effectiveness, efficiency, feasibility, and relevance of these interventions in terms of context and culture. These approaches inherently focus on the target population’s needs, preferences, perspectives, and experiences [80]. Consequently, these interventions are more likely to address health outcomes effectively and foster a sense of ownership. This sense of ownership has been demonstrated to not only increase the acceptability and sustainability of the interventions but also enhance engagement and participation [81].

The successful implementation and effectiveness of digital nutrition interventions in LMICs are often closely linked to the involvement of various healthcare professionals, such as dietitians and community health workers. Their involvement can range from direct counseling via telehealth platforms and remote monitoring to providing technical support or integrating digital tools into routine clinical practice [82]. Study indicates that healthcare professionals generally view digital interventions as valuable tools for extending their reach, improving patient self-management and engagement, and supporting behavior change [83]. Engaging healthcare professionals in the design and implementation process, as well as providing targeted training and support, are essential strategies for maximizing the impact, successful adoption, and sustainability of digital health interventions [84]. Future research should further explore the perspectives and needs of healthcare professionals to inform the development of user-friendly, context-appropriate digital solutions in LMICs.

Nutrition interventions often have a narrow focus, typically targeting specific nutrition behaviours or nutritional deficiencies rather than adopting a comprehensive approach, which may limit their overall impact. They are also often addressed through segregated strategies, leading to fragmented intervention efforts. Our review reflects this phenomenon, with most studies focusing on single aspects of nutrition and few addressing nutritional indicators such as macro- and micronutrient intake and anaemia as outcomes. Double-duty action strategies, for example, are designed to concurrently address undernutrition, obesity, and diet-related NCDs [85]. These strategies are particularly relevant to implement in countries experiencing nutritional transitions, as is the case for many LMICs. A comprehensive focus on nutritional interventions is advantageous due to its potential to effectively address complex and multifactorial nutritional issues, especially within vulnerable populations, including women, children, and the elderly. The necessity for nutritional interventions to encompass a broader range of actions that integrate both nutrition-specific and nutrition-sensitive efforts is emphasized, ultimately enhancing their efficacy and reach across diverse contexts [86]. Furthermore, expanding interventions to target both the immediate and underlying causes of nutritional problems could significantly amplify their impact by addressing social, economic, and environmental factors [87].

Nonetheless, challenges persist in the implementation of digital interventions. For instance, it is often challenging to ascertain the specific impact of text messaging strategies in certain studies, as SMS is frequently employed as a supplementary component alongside other intervention delivery methods, such as websites, emails, or face-to-face communication (multicomponent), rather than a comprehensive strategy [88]. Our review also identified that numerous studies employing multiple delivery platforms and multicomponent strategies exhibited inconsistent results regarding the intervention's impact on outcomes. This suggests that utilizing multiple delivery strategies does not necessarily yield better outcomes compared to a "standalone" strategy. The study also reported that some challenges when implementing digital intervention are common in LMICs, including inadequate internet coverage, limited familiarity with mobile software or devices, and low levels of digital literacy and competency among users [16]. Furthermore, digital interventions may not be feasible in emergency situations or with populations that are difficult to reach, as multiple contacts necessary for troubleshooting potential issues during the intervention may not be possible [16]. Shorter intervention durations, which range from a few weeks to a couple of months in some studies, along with small sample sizes, may plausibly explain the inconclusive findings and may have been insufficient to produce measurable changes in anthropometric or body composition outcomes. Consequently, it is essential to conduct needs assessments, employ intervention mapping approaches, and undertake formative research prior to intervention. This approach is crucial for facilitating the adoption and scaling up of digital health interventions, as it necessitates an understanding of digital literacy levels, which subsequently affects the usability of a digital health tool [89]. Additionally, future digital nutrition interventions in LMICs should explore the integration of artificial intelligence (AI) to enable a more personalized and adaptive experience for users. AI-powered features, such as chatbots for tailored nutrition advice, dietary recommendations, and adaptive interventions in real time, make nutrition guidance more accurate, engaging, and understandable [90].

The review presented several limitations. Firstly, we limited inclusion to English-language publications to ensure consistency in data extraction and summary, thus potentially excluding studies conducted in developing countries that are not published in English. However, only one record was excluded for language, which suggests that this criterion had minimal impact on the comprehensiveness of our review. Secondly, the majority of the identified studies originated from Asia and upper-middle-income countries, which complicates the generalization of findings across LMICs. Thirdly, the diverse nature of study designs, interventions, outcomes, and study samples rendered a meta-analysis and the drawing of definitive conclusions unfeasible within the scoping review approach. A focused or targeted systematic review and meta-analysis on the efficacy of digital nutrition interventions for specific age groups or health outcomes in LMIC settings would be valuable. Despite these limitations, a notable strength of this review is the extensive search and summary of a large number of information sources to identify relevant studies. Furthermore, the results of the included studies, both quantitative and qualitative, were extracted in detail, notwithstanding the complexity of the studies.

## Conclusion

In conclusion, our scoping review identified evidence of digital nutrition interventions implemented in LMICs, with only a limited number of studies focusing on nutrient intake and anaemia. Future research should expand to diverse regions and underrepresented regions—such as Africa, integrate theory-driven and local context-specific approaches that incorporate comprehensive needs assessments, and adopt double-duty action strategies to enhance the effectiveness and reach of digital nutrition interventions in LMIC settings where evidence remains scarce. Moreover, integrating continuous monitoring and support mechanisms, including feedback loops and social support, may further enhance the effectiveness of the interventions. Expanding research into integrating advanced digital technologies like AI will also be beneficial to advancing the field. Future primary research should also explore the feasibility and acceptability of these interventions from both perspectives (users and implementers), which were often underexamined in the existing literature. As digital nutrition interventions become increasingly prevalent globally, particularly among younger people, it is important to consider that digital nutrition technologies are not regarded as replacements for traditional intervention methods. Instead, digital interventions should be viewed as one of several strategies to achieve universal health for the population. To this end, it is essential for local institutions, organizations, stakeholders, and implementers to rigorously address the limitations of current digital interventions. This can be accomplished by actively engaging target participants to enhance their digital health literacy and involving them in the co-design of digital nutrition intervention developments (user-centered design). Ultimately, this scoping review serves as a crucial stepping stone and lays the groundwork for more rigorous studies. It provides a roadmap for researchers, policymakers, and practitioners to build on current evidence, address knowledge gaps, mitigate the challenges, and maximize the public health impact of digital health strategies.

## Supplementary Information

Additional file1 (DOCX 28 KB)

## Data Availability

No datasets were generated or analysed during the current study.
