# Supplementary material for: Digital intervention targeting nutrition and physical activity behaviours among healthy individuals in low- and middle-income countries: a scoping review
Source: J Health Popul Nutr. 2025 Oct 3;44:348. doi: 10.1186/s41043-025-01091-y (PMC12492931; doi:10.1186/s41043-025-01091-y)
Supplement: Supplementary file 1 — Additional file1 (DOCX 28 KB) [file 41043_2025_1091_MOESM1_ESM.docx]

**Supplementary Table 1.** Search strategies for scoping review

| **No** | **PCC model** | **Pubmed Search terms** |
| --- | --- | --- |
| #1 | **Participant** | |
|  | Low- and middle-income countries (LMICs) | (Afghanistan*[tiab] OR Albania*[tiab] OR Algeria*[tiab] OR Samoa*[tiab] OR Angola*[tiab] OR Armenia*[tiab] OR Azerbaijan*[tiab] OR Bangladesh*[tiab] OR Bengali[tiab] OR Belarus*[tiab] OR Belize[tiab] OR Benin[tiab] OR Bhutan*[tiab] OR Bolivia*[tiab] OR Bosnia*[tiab] OR Herzegovina*[tiab] OR Botswana*[tiab] OR Brazil*[tiab] OR Bulgaria*[tiab] OR “Burkina Faso”[tiab] OR Burkinabe[tiab] OR Burundi*[tiab] OR “Cabo Verd*”[tiab] OR “Cape Verd*”[tiab] OR Cambodia*[tiab] OR Cameroon*[tiab] OR “Central African*”[tiab] OR Chad*[tiab] OR China[tiab] OR Chinese[tiab] OR Colombia*[tiab] OR Comoros[tiab] OR Congo[tiab] OR “Costa Rica*”[tiab] OR “Cote d'Ivoire”[tiab] OR “Ivory Coast”[tiab] OR Cuba[tiab] OR Cuban[tiab] OR Djibouti[tiab] OR Dominica*[tiab] OR Ecuador[tiab] OR Egypt*[tiab] OR “El Salvador*”[tiab] OR Eritrea*[tiab] OR Ethiopia*[tiab] OR Fiji*[tiab] OR Gabon*[tiab] OR Gambia*[tiab] OR Georgia*[tiab] OR Ghana*[tiab] OR Grenada*[tiab] OR Guatemala*[tiab] OR Guinea*[tiab] OR Guyan*[tiab] OR Haiti*[tiab] OR Hondura*[tiab] OR India[tiab] OR Indian*[tiab] OR Indonesia*[tiab] OR Iran*[tiab] OR Iraq*[tiab] OR Jamaica*[tiab] OR Jordan*[tiab] OR Kazakh*[tiab] OR Kenya*[tiab] OR Kiribati[tiab] OR “People's Republic of Korea”[tiab] OR “North Korea”[tiab] OR Kosovo[tiab] OR Kosovar*[tiab] OR Kyrgyz*[tiab] OR Lao[tiab] OR Laos[tiab] OR Laotian*[tiab] OR Lebanon[tiab] OR Lebanes*[tiab] OR Lesotho[tiab] OR Liberia*[tiab] OR Libya*[tiab] OR Macedonia*[tiab] OR Madagascar*[tiab] OR Malawi*[tiab] OR Malaysia*[tiab] OR Maldives[tiab] OR Mali[tiab] OR “Marshall Island*”[tiab] OR “Mexico”[MeSH] OR Mexico[tiab] OR Mexican*[tiab] OR Micronesia*[tiab] OR Moldova*[tiab] OR Mongolia*[tiab] OR Montenegr*[tiab] OR Morocc*[tiab] OR Mozambique[tiab] OR Myanmar[tiab] OR Burmese*[tiab] OR Burma[tiab] OR Namibia*[tiab] OR Nepal*[tiab] OR Nicaragua*[tiab] OR Niger*[tiab] OR Pakistan*[tiab] OR Paraguay*[tiab] OR Peru*[tiab] OR Philippin*[tiab] OR Rwanda*[tiab] OR “Sao Tome”[tiab] OR Principe[tiab] OR Senegal*[tiab] OR Serbia*[tiab] OR “Sierra Leone*”[tiab] OR “Solomon Island*”[tiab] OR Somalia*[tiab] OR “South Africa*”[tiab] OR “Sri Lanka”[tiab] OR “St Lucia”[tiab] OR “Saint Lucia”[tiab] OR “St Vincent”[tiab] OR “Saint Vincent”[tiab] OR Grenad*[tiab] OR Sudan*[tiab] OR Suriname*[tiab] OR Swaziland*[tiab] OR Eswatini*[tiab] OR Syria*[tiab] OR Tajik*[tiab] OR Tanzania*[tiab] OR Zanzibar[tiab] OR Thai*[tiab] OR Timor*[tiab] OR Togo*[tiab] OR Tonga*[tiab] OR Tunisia*[tiab] OR Turkey[tiab] OR Turkish[tiab] OR Turkmen*[tiab] OR Tuvalu*[tiab] OR Uganda*[tiab] OR Ukrain*[tiab] OR Uzbeki*[tiab] OR Vanuatu*[tiab] OR Venezuela*[tiab] OR Vietnam*[tiab] OR “Viet nam*”[tiab] OR “West Bank”[tiab] OR Gaza*[tiab] OR Palestin*[tiab] OR Yemen*[tiab] OR Zambia*[tiab] OR Zimbabw*[tiab] OR “Western Sahara”[tiab] OR Argentin*[tiab] OR Russia*[tiab] OR Maurit*[tiab] OR Palau[tiab] OR Romania*[tiab]) |
| #2 | **Concept** | |
|  | Nutrition and physical activity intervention | ("Health Education"[Mesh:NoExp] OR "Health Promotion"[Mesh]) OR ("Diet, Healthy"[Mesh] OR healthy diet*[tiab] OR healthy eating[tiab] OR healthy food*[tiab] OR diet education[tiab] OR dietary education[tiab] OR dietary intervention[tiab] OR dietary therapy*[tiab] OR diet restriction*[tiab] OR healthy nutrition*[tiab] OR diet application*[tiab] OR weight loss[tiab] OR calorie control*[tiab] OR nutrition counsel*[tiab] OR nutritional counsel*[tiab]) OR (nutrition education[tiab] OR nutrition intervention[tiab]) OR ("exercise"[MeSH] OR "exercise" [tiab] OR "physical activity"[tiab] OR fitness*[tiab] OR sport*[tiab]) |
| #3 | **Context** | |
|  | Digital intervention | ("Mobile Applications"[Mesh] OR “digital app*”[tiab] OR “smartphone app*”[tiab] OR “mobile app*”[tiab] OR “social media”[tiab] OR "Telemedicine"[Mesh] OR "telemedicine"[tiab] OR "telehealth" [tiab] OR "tele-health"[tiab] OR ehealth[tiab] OR "e-health"[tiab] OR "mhealth"[tiab] OR "m-health"[tiab] OR “digital platform”[tiab] OR “internet platform”[tiab] OR “online platform”[tiab] OR “mobile nutrition”[tiab] OR “nutrition application*”[tiab] OR e-learning nutrition[tiab] OR “telenutrition”[tiab] OR ehealth nutrition[tiab] OR “enutrition”[tiab]) OR (“exergaming”[Mesh] OR “exergame*”[tiab] OR “video games”[Mesh] OR “video game*”[tiab] OR “gamification”[Mesh] OR gamification[tiab]) |
| #4 | **Type of Study** | |
|  |  | ("randomized controlled trial"[pt] OR "random allocation"[mesh] OR "cross-over studies"[mesh] OR "Controlled Before-After Studies"[Mesh] OR quasi experiment*[tiab] OR quasiexperiment*[tiab] OR “quasi-experiment*”[tiab]) |
| **No** | **PCC model** | **Embase Search terms** |
| #1 | **Participant** | |
|  | Low- and middle-income countries (LMICs) | (Afghanistan*:ti,ab OR Albania*:ti,ab OR Algeria*:ti,ab OR Samoa*:ti,ab OR Angola*:ti,ab OR Armenia*:ti,ab OR Azerbaijan*:ti,ab OR Bangladesh*:ti,ab OR Bengali:ti,ab OR Belarus*:ti,ab OR Belize:ti,ab OR Benin:ti,ab OR Bhutan*:ti,ab OR Bolivia*:ti,ab OR Bosnia*:ti,ab OR Herzegovina*:ti,ab OR Botswana*:ti,ab OR Brazil*:ti,ab OR Bulgaria*:ti,ab OR ‘Burkina Faso’:ti,ab OR Burkinabe:ti,ab OR Burundi*:ti,ab OR ‘Cabo Verd*’:ti,ab OR ‘Cape Verd*’:ti,ab OR Cambodia*:ti,ab OR Cameroon*:ti,ab OR ‘Central African*’:ti,ab OR Chad*:ti,ab OR China:ti,ab OR Chinese:ti,ab OR Colombia*:ti,ab OR Comoros:ti,ab OR Congo:ti,ab OR ‘Costa Rica*’:ti,ab OR ‘cote d`ivoire’:ti,ab OR ‘Ivory Coast’:ti,ab OR Cuba:ti,ab OR Cuban:ti,ab OR Djibouti:ti,ab OR Dominica*:ti,ab OR Ecuador:ti,ab OR Egypt*:ti,ab OR ‘El Salvador*’:ti,ab OR Eritrea*:ti,ab OR Ethiopia*:ti,ab OR Fiji*:ti,ab OR Gabon*:ti,ab OR Gambia*:ti,ab OR Georgia*:ti,ab OR Ghana*:ti,ab OR Grenada*:ti,ab OR Guatemala*:ti,ab OR Guinea*:ti,ab OR Guyan*:ti,ab OR Haiti*:ti,ab OR Hondura*:ti,ab OR India:ti,ab OR Indian*:ti,ab OR Indonesia*:ti,ab OR Iran*:ti,ab OR Iraq*:ti,ab OR Jamaica*:ti,ab OR Jordan*:ti,ab OR Kazakh*:ti,ab OR Kenya*:ti,ab OR Kiribati:ti,ab OR ‘People`s Republic of Korea’:ti,ab OR ‘North Korea’:ti,ab OR Kosovo:ti,ab OR Kosovar*:ti,ab OR Kyrgyz*:ti,ab OR Lao:ti,ab OR Laos:ti,ab OR Laotian*:ti,ab OR Lebanon:ti,ab OR Lebanes*:ti,ab OR Lesotho:ti,ab OR Liberia*:ti,ab OR Libya*:ti,ab OR Macedonia*:ti,ab OR Madagascar*:ti,ab OR Malawi*:ti,ab OR Malaysia*:ti,ab OR Maldives:ti,ab OR Mali:ti,ab OR ‘Marshall Island*’:ti,ab OR 'Mexico'/exp OR Mexico:ti,ab OR Mexican*:ti,ab OR Micronesia*:ti,ab OR Moldova*:ti,ab OR Mongolia*:ti,ab OR Montenegr*:ti,ab OR Morocc*:ti,ab OR Mozambique:ti,ab OR Myanmar:ti,ab OR Burmese*:ti,ab OR Burma:ti,ab OR Namibia*:ti,ab OR Nepal*:ti,ab OR Nicaragua*:ti,ab OR Niger*:ti,ab OR Pakistan*:ti,ab OR Paraguay*:ti,ab OR Peru*:ti,ab OR Philippin*:ti,ab OR Rwanda*:ti,ab OR ‘Sao Tome”:ti,ab OR Principe:ti,ab OR Senegal*:ti,ab OR Serbia*:ti,ab OR ‘Sierra Leone*’:ti,ab OR ‘Solomon Island*’:ti,ab OR Somalia*:ti,ab OR ‘South Africa*’:ti,ab OR ‘Sri Lanka’:ti,ab OR ‘St Lucia’:ti,ab OR ‘Saint Lucia’:ti,ab OR ‘St Vincent’:ti,ab OR ‘Saint Vincent’:ti,ab OR Grenad*:ti,ab OR Sudan*:ti,ab OR Suriname*:ti,ab OR Swaziland*:ti,ab OR Eswatini*:ti,ab OR Syria*:ti,ab OR Tajik*:ti,ab OR Tanzania*:ti,ab OR Zanzibar:ti,ab OR Thai*:ti,ab OR Timor*:ti,ab OR Togo*:ti,ab OR Tonga*:ti,ab OR Tunisia*:ti,ab OR Turkey:ti,ab OR Turkish:ti,ab OR Turkmen*:ti,ab OR Tuvalu*:ti,ab OR Uganda*:ti,ab OR Ukrain*:ti,ab OR Uzbeki*:ti,ab OR Vanuatu*:ti,ab OR Venezuela*:ti,ab OR Vietnam*:ti,ab OR ‘Viet nam*’:ti,ab OR ‘West Bank’:ti,ab OR Gaza*:ti,ab OR Palestin*:ti,ab OR Yemen*:ti,ab OR Zambia*:ti,ab OR Zimbabw*:ti,ab OR ‘Western Sahara’:ti,ab OR Argentin*:ti,ab OR Russia*:ti,ab OR Maurit*:ti,ab OR Palau:ti,ab OR Romania*:ti,ab) |
| #2 | **Concept** | |
|  | Nutrition and physical activity intervention | ('health education'/de OR 'health promotion'/exp) OR ('preventive health service'/de) OR (preventive health:ti,ab) OR ('healthy diet'/exp OR healthy diet*:ti,ab OR healthy eating:ti,ab OR healthy food*:ti,ab OR diet education:ti,ab OR dietary intervention:ti,ab OR diet therapy*:ti,ab OR diet restriction*: ti,ab OR body weight loss: ti,ab OR calorie control*:ti,ab OR diet application*:ti,ab OR healthy nutrition*:ti,ab OR nutrition counsel*:ti,ab OR nutritional counsel*:ti,ab) OR (nutrition education:ti,ab OR nutrition intervention:ti,ab) OR ('exercise'/exp OR 'exercise':ti,ab OR 'physical activity':ti,ab OR fitness*:ti,ab OR sport*:ti,ab) |
| #3 | **Context** | |
|  | Digital intervention | ('mobile application'/exp OR 'mobile health application'/exp OR 'telehealth'/exp OR 'telemedicine'/exp OR 'social media'/exp) OR (telemedicine:ti,ab OR telehealth:ti,ab OR 'mobile health application':ti,ab OR 'digital app*':ti,ab OR 'smartphone app*':ti,ab OR 'mobile app*':ti,ab OR 'social media':ti,ab OR ehealth:ti,ab OR e-health:ti,ab OR mhealth:ti,ab OR m-health:ti,ab OR 'digital platform':ti,ab OR 'internet platform':ti,ab OR 'online platform':ti,ab OR 'mobile nutrition':ti,ab OR 'nutrition application*':ti,ab OR 'elearning nutrition':ti,ab OR 'e-learning nutrition':ti,ab OR telenutrition:ti,ab OR 'ehealth nutrition':ti,ab OR enutrition:ti,ab) OR ('exergaming'/exp OR exergame*:ti,ab OR 'video game'/exp OR 'video game*':ti,ab OR 'gamification'/exp OR gamification:ti,ab) |
| #4 | **Type of study** | |
|  |  | ('randomized controlled trial':af OR 'randomization'/exp OR 'crossover procedure'/exp OR 'epidemiology'/exp OR quasi experiment*:ti,ab OR quasiexperiment*:ti,ab OR 'quasi-experiment*':ti,ab) |
| **No** | **PCC model** | **Cochrane Search terms** |
| #1 | **Participant** | |
|  | Low- and middle-income countries (LMICs) | (Afghanistan*):ti,ab,kw OR (Albania*):ti,ab,kw OR (Algeria*):ti,ab,kw OR (Samoa*):ti,ab,kw OR (Angola*):ti,ab,kw OR (Armenia*):ti,ab,kw OR (Azerbaijan*):ti,ab,kw OR (Bangladesh*):ti,ab,kw OR (Bengali):ti,ab,kw OR (Belarus*):ti,ab,kw OR (Belize):ti,ab,kw OR (Benin):ti,ab,kw OR (Bhutan*):ti,ab,kw OR (Bolivia*):ti,ab,kw OR (Bosnia*):ti,ab,kw OR (Herzegovina*):ti,ab,kw OR (Botswana*):ti,ab,kw OR (Brazil*):ti,ab,kw OR (Bulgaria*):ti,ab,kw OR ("Burkina Faso"):ti,ab,kw OR (Burkinabe):ti,ab,kw OR (Burundi*):ti,ab,kw OR (Cabo NEXT Verd*):ti,ab,kw OR (Cape NEXT Verd*):ti,ab,kw OR (Cambodia*):ti,ab,kw OR (Cameroon*):ti,ab,kw OR (Central African*):ti,ab,kw OR (Chad*):ti,ab,kw OR (China):ti,ab,kw OR (Chinese):ti,ab,kw OR (Colombia*):ti,ab,kw OR (Comoros):ti,ab,kw OR (Congo):ti,ab,kw OR ("Cook Islands"):ti,ab,kw OR (Costa NEXT Rica*):ti,ab,kw OR ("Cote d'Ivoire"):ti,ab,kw OR ("Ivory Coast"):ti,ab,kw OR (Cuba):ti,ab,kw OR (Cuban):ti,ab,kw OR (Djibouti):ti,ab,kw OR (Dominica*):ti,ab,kw OR (Ecuador):ti,ab,kw OR (Egypt):ti,ab,kw OR (El NEXT Salvador*):ti,ab,kw OR (Eritrea*):ti,ab,kw OR (Ethiopia*):ti,ab,kw OR (Fiji*):ti,ab,kw OR (Gabon*):ti,ab,kw OR (Gambia*):ti,ab,kw OR (Georgia*):ti,ab,kw OR (Ghana*):ti,ab,kw OR (Grenada*):ti,ab,kw OR (Guadeloupe):ti,ab,kw OR (Guatemala*):ti,ab,kw OR (Guinea*):ti,ab,kw OR (Guyan*):ti,ab,kw OR (Haiti*):ti,ab,kw OR (Hondura*):ti,ab,kw OR (India):ti,ab,kw OR (Indian*):ti,ab,kw OR (Indonesia*):ti,ab,kw OR (Iran*):ti,ab,kw OR (Iraq*):ti,ab,kw OR (Jamaica*):ti,ab,kw OR (Jordan*):ti,ab,kw OR (Kazakh*):ti,ab,kw OR (Kenya*):ti,ab,kw OR (Kiribati):ti,ab,kw OR ("People's Republic of Korea"):ti,ab,kw OR ("North Korea"):ti,ab,kw OR (Kosovo):ti,ab,kw OR (Kosovar*):ti,ab,kw OR (Kyrgyz*):ti,ab,kw OR (Lao):ti,ab,kw OR (Laos):ti,ab,kw OR (Laotian*):ti,ab,kw OR (Lebanon):ti,ab,kw OR (Lebanes*):ti,ab,kw OR (Lesotho):ti,ab,kw OR (Liberia*):ti,ab,kw OR (Libya*):ti,ab,kw OR (Macedonia*):ti,ab,kw OR (Madagascar*):ti,ab,kw OR (Malawi*):ti,ab,kw OR (Malaysia*):ti,ab,kw OR (Maldives):ti,ab,kw OR (Mali):ti,ab,kw OR (Marshall NEXT Island*):ti,ab,kw OR [mh Mexico] OR (Mexico):ti,ab,kw OR (Mexican*):ti,ab,kw OR (Micronesia*):ti,ab,kw OR (Moldova*):ti,ab,kw OR (Mongolia*):ti,ab,kw OR (Montenegr*):ti,ab,kw OR (Morocc*):ti,ab,kw OR (Mozambique):ti,ab,kw OR (Myanmar):ti,ab,kw OR (Burmese*):ti,ab,kw OR (Burma):ti,ab,kw OR (Namibia*):ti,ab,kw OR (Nepal*):ti,ab,kw OR (Nicaragua*):ti,ab,kw OR (Niger*):ti,ab,kw OR (Pakistan*):ti,ab,kw OR (Paraguay*):ti,ab,kw OR (Peru*):ti,ab,kw OR (Philippin*):ti,ab,kw OR (Rwanda*):ti,ab,kw OR ("Sao Tome"):ti,ab,kw OR (Principe):ti,ab,kw OR (Senegal*):ti,ab,kw OR (Serbia*):ti,ab,kw OR (Sierra NEXT Leone*):ti,ab,kw OR (Solomon NEXT Island*):ti,ab,kw ОR (Somalia*):ti,ab,kw OR (South NEXT Africa*):ti,ab,kw OR ("Sri Lanka"):ti,ab,kw OR ("St Lucia"):ti,ab,kw OR ("Saint Lucia"):ti,ab,kw OR ("St Vincent"):ti,ab,kw OR ("Saint Vincent"):ti,ab,kw OR (Grenad*):ti,ab,kw OR (Sudan*):ti,ab,kw OR (Suriname*):ti,ab,kw OR (Swaziland*):ti,ab,kw OR (Eswatini*):ti,ab,kw OR (Syria*):ti,ab,kw OR (Tajik*):ti,ab,kw OR (Tanzania*):ti,ab,kw OR (Zanzibar):ti,ab,kw OR (Thai*):ti,ab,kw OR (Timor*):ti,ab,kw OR (Togo*):ti,ab,kw OR (Tonga*):ti,ab,kw OR (Tunisia*):ti,ab,kw OR (Turkey):ti,ab,kw OR (Turkish):ti,ab,kw OR (Turkmen*):ti,ab,kw OR (Tuvalu*):ti,ab,kw OR (Uganda*):ti,ab,kw OR (Ukrain*):ti,ab,kw OR (Uzbeki*):ti,ab,kw OR (Vanuatu*):ti,ab,kw OR (Venezuela*):ti,ab,kw OR (Vietnam*):ti,ab,kw OR (Viet NEXT nam*):ti,ab,kw OR ("West Bank"):ti,ab,kw OR (Gaza*):ti,ab,kw OR (Palestin*):ti,ab,kw OR (Yemen*):ti,ab,kw OR (Zambia*):ti,ab,kw OR (Zimbabw*):ti,ab,kw OR ("Western Sahara"):ti,ab,kw OR (Argentin*):ti,ab,kw OR (Russia*):ti,ab,kw OR (Maurit*):ti,ab,kw OR (Palau):ti,ab,kw OR (Romania*):ti,ab,kw) |
| #2 | **Concept** | |
|  | Nutrition and physical activity intervention | [mh ^"health education"] OR [mh ^"health promotion"] OR [mh ^"preventive health service"] OR ("preventive health behavior"):ti,ab,kw OR (preventive NEXT health NEXT behaviour*):ti,ab,kw OR (preventive NEXT health):ti,ab,kw OR [mh "diet, healthy"] OR (healthy NEXT diet*):ti,ab,kw OR (healthy NEXT eating):ti,ab,kw OR (healthy NEXT food*):ti,ab,kw OR (dietary NEXT education):ti,ab,kw OR (dietary NEXT intervention):ti,ab,kw OR (diet NEXT therapy*):ti,ab,kw OR (diet NEXT restriction*):ti,ab,kw OR (body NEXT weight NEXT loss):ti,ab,kw OR (calorie NEXT control*):ti,ab,kw OR (diet NEXT application*):ti,ab,kw OR (healthy NEXT nutrition*):ti,ab,kw OR (nutrition NEXT counsel*):ti,ab,kw OR (nutritional NEXT counsel*):ti,ab,kw OR (nutrition NEXT education):ti,ab,kw OR (nutrition NEXT intervention):ti,ab,kw OR [mh "exercise"] OR ("exercise"):ti,ab,kw OR ("physical NEXT activity"):ti,ab,kw OR (fitness*):ti,ab,kw OR (sport*):ti,ab,kw |
| #3 | **Context** | |
|  | Digital intervention | [mh ^"mobile applications"] OR [mh ^"telemedicine"] OR [mh "social media"] OR (mobile NEXT health NEXT application):ti,ab,kw OR ("telehealth"):ti,ab,kw OR (telemedicine):ti,ab,kw OR (social NEXT media):ti,ab,kw OR (digital NEXT app*):ti,ab,kw OR (smartphone NEXT app*):ti,ab,kw OR (mobile NEXT app*):ti,ab,kw OR (ehealth):ti,ab,kw OR (e-health):ti,ab,kw OR (mhealth):ti,ab,kw OR (m-health):ti,ab,kw OR (digital NEXT platform):ti,ab,kw OR (internet NEXT platform):ti,ab,kw OR (online NEXT platform):ti,ab,kw OR (mobile NEXT nutrition):ti,ab,kw OR (nutrition NEXT app*):ti,ab,kw OR (elearning NEXT nutrition):ti,ab,kw OR (e-learning NEXT nutrition):ti,ab,kw OR (telenutrition):ti,ab,kw OR (ehealth NEXT nutrition):ti,ab,kw OR (enutrition):ti,ab,kw OR [mh "exergaming"] OR [mh "video games"] OR [mh "gamification"] OR (exergame*):ti,ab,kw OR (video NEXT game*):ti,ab,kw OR (gamification):ti,ab,kw |
| #4 | **Type of study** | |
|  |  | [mh ^"randomized controlled trial"] OR [mh "random allocation"] OR [mh ^"cross-over studies"] OR [mh ^"Controlled Before-After Studies"] OR [mh ^"non-randomized controlled trial as topic"] OR (quasi NEXT experiment*):ti,ab OR quasiexperiment*:ti,ab OR (quasi-experiment*):ti,ab |

**Supplementary Table 2.** List of excluded articles during full-text review

| **No** | **Title** | **Author, year** | **Country** | **Subjects** | **Reason** |
| --- | --- | --- | --- | --- | --- |
| 1 | Influence of counseling applications on improving the balancing nutritional knowledge for pregnant women | B 2021 | Indonesia | Healthy pregnant women | Exclude, not enough information |
| 2 | Effectiveness of social media with or without wearable devices to improve physical activity and reduce sedentary behavior: A randomized controlled trial of Chinese postgraduates | Li 2023 | China | Healthy adults | Exclude, App is not as main intervention platform; both groups received social media intervention - the different only intervention group use wearable devices |
| 3 | The Use of Media in Stunting Extension to Adolescents at Senior High School, Langsa City | Lina 2022 | Indonesia | Healthy youth | Exclude, not enough information |
| 4 | Can Dietary Instructions Delivered Through Mobile Application Reduce Sweet Score among Adolescents in Chennai, India?--A Randomized Controlled Preventive Trial | Sivasamy 2020 | India | Healthy adolescents | Exclude, not enough information |
| 5 | Impact of a group-based intervention program on physical activity and health-related outcomes in worksite settings | Gu 2020 | China | Healthy adults | Exclude, App is not as main intervention platform |
| 6 | The Smartphone-Assisted Intervention Improved Perception of Nutritional Status among Middle School Students | Shen 2020 | China | Healthy adolescents | Exclude, App is not as main intervention platform, just to input BMI and researcher can provide feedback |
| 7 | Effect of Exergames on Physical Function, Cognitive Capacity, Depressive State and Fall-Risk in Mexican Older Adults: A pilot study | Gomez-miranda 2019 | Mexico | Healthy older adults | Exclude, App is not as main intervention platform |
